# Isocitrate dehydrogenase 1 mutation subtypes at site 132 and their translational potential in glioma

**DOI:** 10.2217/cns-2017-0019

**Published:** 2018-01-05

**Authors:** Victor M Lu, Kerrie L McDonald

**Affiliations:** 1 Cure Brain Cancer Neuro-oncology Laboratory, Prince of Wales Clinical School, Lowy Cancer Research Centre, University of New South Wales, Sydney, Australia

**Keywords:** glioma, isocitrate dehydrogenase, R132H, mutation subtype

## Abstract

In recent years, *de novo* missense structural mutations in the *IDH1* gene of arginine at site 132 (R132) have become a standard for diagnostication and prognostication in glioma management. As our clinical understanding of this mutation grows, so too does the number of mutation subtypes reported in the literature. By synergizing current knowledge of IDH1 activity in glioma with the emerging evidence of different enzyme kinetics between R132 *IDH1* mutation subtypes, the translational potential in improving glioma management based on mutated *IDH1* subtype in glioma is described.

**Practice points**

- Mutated isocitrate dehydrogenase 1 (mIDH1) confers a greater survival benefit in glioma.
- Approximately 90% of this mutation is R132H (arginine to histidine mutation at site 132) subtype in lower-grade glioma, and the remaining 10% is made up of other nonhistidine amino acid (AA) substitutions.
- The selection of mIDH1 subtype may be a result of complex interplay with other genetic changes and glia type in glioma.
- Wild-type isocitrate dehydrogenase 1 (wtIDH1) primarily functions to convert isocitrate (ICT) to α-ketoglutarate (αKG) in the cytosol as part of the tricarboxylic acid cycle.
- wtIDH1 also serves to restore glutathione which provides the cell with protection from radical oxygen species.
- mIDH1 results in the additional conversion of αKG to D-2-hydroxyglutarate (D2HG), which is likely an oncometabolite.
- The depletion of αKG leads to increased glutaminolysis.
- In wtIDH1, the arginine at site 132 is required to coordinate the proper binding of ICT at the active site.
- In mIDH1, the AA substitution of arginine results in disrupted ICT coordination at the active site leading to changes in enzyme kinetics.
- Different substituting AAs in mIDH1 lead to different enzyme kinetics and rates of production of D2HG.
- AA properties such as hydrophobicity and residue size are demonstrated influential factors.
- D2HG can be used as an indicator for glioma prognosis and its relative production can be predicted based on mIDH1 subtype and it is substituting AA.
- AA properties to consider hydrophobicity, van der Waal volumes, size and bonding interactions.
- Preclinical trials inhibiting mIDH1 are promising; however, results of current ongoing clinical trials will reveal how effective mIDH1 is as a potential therapeutic target.
- Current ‘The Cancer Genome Atlas’ data indicate that not all mIDH1 subtypes confer a survival benefit; however, these results are severely limited.
- Dissimilarity of substituting AA to arginine, of the R132 wtIDH1, may signal how treatable the glioma is based on superior outcomes associated with mIDH1.

The discovery of *de novo* mutations in the *IDH* gene (*EC1.1.1.*42) has revolutionized glioma management. A large body of evidence suggests that the detection of mutated IDH (mIDH) in glioma affords a survival advantage compared with wild-type IDH (wtIDH) [1–3]. The exact causative mechanisms remain uncertain; however, recent evidence suggests increased chemosensitivity [4], epigenetic changes [5] and increase in methylation [6] are likely to be involved. Mutations in cytosolic *IDH1* isoform occur in approximately 80% of all lower-grade glioma (LGG) and secondary glioblastoma [3]. The majority of mIDH1 subtypes derive from missense substitutions of arginine (R, Arg) at codon site 132 (R132). The most common subtype in all glioma is R132H, a histidine (H, His) substitution, and makes up to approximately 90% of all LGG mIDH1 subtypes [3,7,8]. Yet, there have been a number of other mIDH1 subtypes which involve other amino acid (AA) R132 substitutions observed in similar glioma populations to date (Table 1).

**Table 1.** **Frequency of reported glioma R132 mutated isocitrate dehydrogenase 1 subtypes in the literature and The Cancer Genome Atlas database concerning lower-grade glioma only.**

**mIDH1 subtype**	**Substituent**	**Frequency of mIDH1 subtype in LGG (%) [3,7–10]**
R132H	Histidine	62–93

R132C	Cysteine	2.9–4.3

R132G	Glycine	1.0–2.5

R132S	Serine	1.1–2.2

R132L	Leucine	0.2–0.6

R132V	Valine	Single cases [11]

R132P	Proline	Single cases [12]

TCGA-LGG results are produced from the publicly available data from the Genomic Data Commons Data Portal accessible at https://portal.gdc.cancer.gov

mIDH1: Mutated isocitrate dehydrogenase 1; R132: Arginine substitution mutation at site 132; TCGA-LGG: The Cancer Genome Atlas lower-grade glioma.

Of particular note is the mIDH1 subtype R132C, a cysteine (C, Cys) substitution, which while not particularly common in general glioma, occurs in all glioma associated with Li-Fraumeni syndrome [13]. Given the exclusiveness of R132C to all Li-Fraumeni glioma, and the hallmark of the syndrome being an established TP53 germline mutation, it is posited that subtype R132C mutation is favored by glioma progenitors when it occurs after a TP53 mutation. Indeed, anecdotal evidence of only mIDH1 R132H subtype occurring, if it occurs before a TP53 mutation in glioma, which appears to be all the cases, except that of Li-Fraumeni, would appear to support this [14]. Furthermore, the same data imply that the IDH1 mutation may in fact be a very early mutative event in gliomagenesis, as another frequent glioma mutation 1p/19q codeletion is not acquired before mIDH1 if they are to copresent. Collectively, current evidence suggests that mIDH1 subtype outcome is, at least partially, dictated by the time point at which it occurs in gliomagenesis relative to other mutations.

It is also a worthy consideration that glioma type and origin may affect mIDH1 subtype, with oligodendroglioma rarely occurring in Li-Fraumeni compared with astrocytoma, and the subtype R132C, which is associated with Li-Fraumeni, rarely occurring in oligodendroglioma compared with astrocytoma overall [13]. This suggests that the mIDH1 R132C subtype may have a lineage predilection for astrocyte cells, or even astrocytoma progenitors with respect to glial cells in general. Additionally, it has been shown *in vitro* that the mIDH1 R132H subtype, at the least, impairs neural stem cell astrogenesis [15]. The effective absence of R132C mutations in oligodendroglioma, which are typically associated with 1p/19q codeletion, indicates that some mIDH1 subtypes may not occur in the presence of other specific genetic changes in glioma [9]. While this requires further study of gliogenic pathways, it is not inconceivable that mIDH1 subtypes may have varying effects on glial progenitor differentiation into different glioma given their different genetic profiles, and vice versa.

From a diagnostic perspective, the presence of mIDH1 specifically appears to favor glioma localization to the right hemisphere [16] and the frontal lobe [17]. However, most likely consequential to the limited sample size of rarer mIDH1 subtypes, there is no evidence to date to suggest further preferential localization based on subtype. This will be important in the future to consider, as it has been suggested that once accounted for location, the prognostic benefit of mIDH1 compared with wtIDH1 may not be so significant in higher grade glioma [18]. Thus, developing a greater understanding of how mIDH1 subtypes compare to each other remains an important goal in furthering our prognostication and management of these gliomas.

Recent biochemistry studies [7,19] have been able to shed light onto the enzymatic differences of mIDH1 R132 subtypes in terms of their cellular function and metabolic effect in glioma. This article aims to draw attention to the translational potential in recognizing these differences in order to continue to optimize glioma management in the future.

## Cellular function

**Figure 1.**
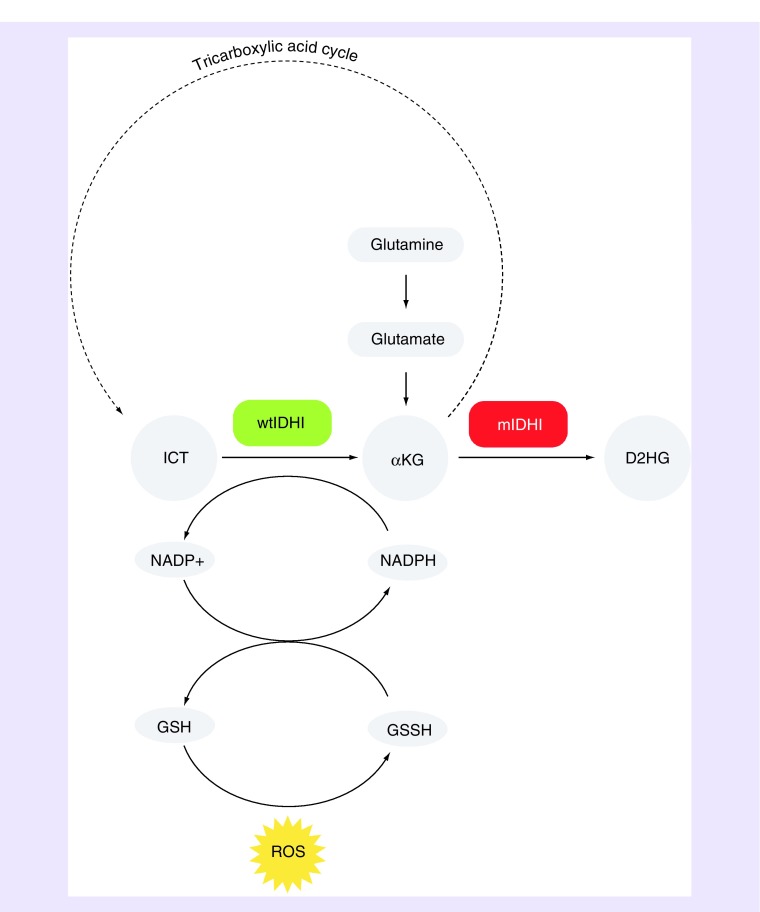
**Schematic summary of the cellular functions of wild-type isocitrate dehydrogenase 1 and mutated isocitrate dehydrogenase 1.** The wtIDH1 functions mainly to catalyze the oxidative conversion of ICT to αKG as part of the TCA cycle. It also functions to restore NADPH from NADP+ in order to regenerate GSH from GSSH to support protection from ROS. The mIDH1 primarily functions to catalyze conversion of αKG to D2HG, depleting the cell of αKG required to complete the TCA cycle. Furthermore, αKG can be produced *via* the breakdown of glutamine, a process termed glutaminolysis, which converts into glutamate first. αKG: α-ketoglutarate; D2HG: D-2-hydroxyglutarate; GSH: Glutathione; GSSH: Glutathione disulfide; ICT: Isocitrate; mIDH1: Mutated isocitrate dehydrogenase 1; NADP+: Nicotinamide adenine dinucleotide phosphate (oxidized); NADPH: Nicotinamide adenine dinucleotide phosphate (reduced); ROS: Radical oxygen species; TCA: Tricarboxylic acid; wtIDH1: Wild-type isocitrate dehydrogenase 1.

IDH1 occurs in the cellular cytosol as part of the tricarboxylic acid (TCA) cycle, and has many functions (Figure 1). As wtIDH1, it primarily acts as a catalyst of the reversible oxidation decarboxylation step of isocitrate (ICT) into α-ketoglutarate (αKG). In this process, nicotinamide adenine dinucleotide phosphate (NAPD^+^, oxidized form) is converted into NADPH (reduced form) which serves as a reconstitutive component of glutathione (GSH) from glutathione disulfide (GSSH) [20]. This is of great importance to cell survival as GSH serves as the inbuilt protection mechanism to neutralize damaging oxidation from radical oxygen species. Additionally, αKG is also the downstream product of glutaminolysis, making it and the wtIDH1 enzyme key components of glutamine and glutamate metabolism within the cell [21,22]. Furthermore, wtIDH1 and its αKG production are involved in a number of cellular metabolic functions, including the modulation of insulin secretion [23] and the promotion of lipogenesis during hypoxia [24].

In the case of mIDH1, the enzyme is less able to facilitate the production of αKG due to structural changes. The consequences of this include reduced cellular glutamine and glutamate due to their metabolism in order to indirectly compensate αKG production, as well as reduced production of antioxidant NADPH which ultimately leads to lower tolerance for oxidative stress. In addition to the loss-of-function of TCA-directed αKG production, mIDH1 affords a gain-of-function neoenzymatic ability to catalyze the reduction of any produced αKG to oncometabolite D-2-hydroxyglutarate (D2HG), an acid with no known physiologic utility [25,26]. This is conceptually a reverse reaction of the (wt)IDH1 forward reaction in that less αKG is produced by normal TCA mechanisms.

Although not certain, the most commonly accepted oncogenic mechanisms of D2HG derive from its structural similarity to that of αKG. They include competitive antagonism of many important αKG-dependent cellular enzymes, primarily dioxygenases such as TET family hydroxylases and JmjC family demethylases resulting in cell dedifferentiation [27], and more controversially, dysregulation of hypoxia-inducible factor 1α expression [28]. Within glioma and progenitor cells, increased D2HG is correlated with increased mitotic activity, apoptosis susceptibility, axonal disruption, vascular neoplasia and with dysregulation of cellular differentiation and several brain metabolites [15,29].

## Enzyme structure

The wtIDH1 enzyme exists as an asymmetric homodimer *in situ* with two active sites per dimer [30]. It is at these active sites where substrate ICT binds to initiate catalysis. It is thought that the initial exposure of the enzyme to cofactor NADP causes the transformation from an inactive and open configuration into an intermediate and inactive configuration. Then, binding by ICT at the active site displaces the regulatory loop segment of the intermediate configuration to transform it into a catalytic, active closed conformation. The affinity of ICT for the active site in the intermediate configuration is mediated by R132, located in the catalytic cleft of the active site. More specifically, the R132 maintains key salt-bridge interactions with the carboxylates of incoming substrate ICT to facilitate forward catalysis [25].

In mIDH1, the R132 is substituted by other AAs. Physically, there is little difference from wtIDH1, with minimal effect on melting temperature and overall geometry of the protein [19]. There is significant effect biochemically however, as the arginine coordination at site 132 required for ICT binding is disrupted, resulting in subsequent configuration disequilibrium with an increase in the intermediate, inactive configuration [3]. This intermediate configuration appears to possess a greater affinity for cofactor NADPH than wtIDH1, which favors the gain-of-function reverse reaction of αKG reduction to D2HG [25]. The genotype of mIDH1 glioma in practice is heterozygous, and the likely presence of both mIDH1 and wtIDH1 subunits within a mutated heterodimer manifests as concurrently occurring forward and reverse IDH1 reactions respectively [31].

## Subtype kinetics

The substituting AAs at R132 in mIDH1 vary greatly in terms of side chain biochemical properties and can affect enzyme activity. Most recently, Matteo *et al*. [19] evaluated the hydrophobicity and van der Waal volumes of substituting AAs in mIDH1 subtypes with respect to D2HG production. They examined *in vitro* the largest number of glioma mIDH1 subtypes in one study to date, three clinical R132H, R132C and R132G; and five engineered R132W, R132A, R132Q, R132K and R132N mutations. They were able to provide independent evidence that in general compared with R132 wtIDH1: less hydrophobic AAs with larger residues favor the forward reaction; and inversely more hydrophobic AAs with smaller residues favor the reverse reaction. While trends were not perfect, this data is the first of its kind to implicate AA properties polarity and size of substituting AAs in various mIDH1 subtypes may predict relative D2HG production efficiencies.

Appreciating the subtle differences in their enzyme kinetics, Pusch *et al*. [7] showed *in vitro* some of the earliest evidence that glioma mIDH1 subtypes differ in terms of their αKG affinities in the reverse IDH1 reaction that produces D2HG. They analyzed the biochemical mIDH1 profiles of R132H, R132C, R132G, R132S and R132L. Of all the mutation subtypes, R132H had the lowest affinity for αKG and lowest production of D2HG. Unfortunately, there was no obvious linear relationship between subtype αKG affinity and D2HG production as while R132L showed the highest affinity for αKG, it was R132G that showed the highest production of D2HG. Nonetheless, it was clear that the substituting AA at R132 in mIDH1 affects αKG affinity which may affect the oncogenic processes associated with D2HG.

## Translational potential

Being able to predict which mIDH1 subtype will produce greater D2HG levels has the potential to influence clinical glioma therapy. If the substituting AA of different subtypes can be stratified into oncogenic potential scales or groups, then upon diagnosis, a clearer prognosis could be declared. This is not unimaginable for the future, given that it was only last year that the WHO declared the diagnosis of brain tumors to require mIDH1 status clarification [32].

Variables of AA to consider could include hydrophobicity, van der Waal volumes, size and bonding interactions. As a result, it would allow clinicians and scientists to stratify various management parameters such as urgency and extent of treatment (surgery, chemotherapy and radiation) and identify glioma mIDH1 subpopulations that may benefit more from the emerging class of IDH1-inhibiting drugs. In addition, appreciating the molecular detail of substituting AAs may allow researchers to develop sterically hindering moieties that are specific to different AA side-chain residues of mIDH1 to inhibit its activity, which would disturb the interaction between αKG and the intermediate, inactive configuration of mIDH1 in an attempt to reduce D2HG conversion.

There has been modest progress made in investigating the inhibition of mIDH1 in glioma to reduce D2HG and retard growth. This concept has been proved *in vitro* with a number of mIDH1 R132H inhibitors, including ML308 [33] and AGI-5198 [34]. To date, the most significant preclinical study remains that by Pusch *et al*. [35], who recently reported very promising results using pan-mIDH1 subtype inhibitor BAY 1436032 with no obvious toxicity *in vitro* or *in vivo*. With this drug, there was significant reduction of D2HG in multiple cancer cell lines carrying mIDH1 subtypes R132H, R132C, R132G, R132S and R132L, including immortalized GBM (LN229) and primary patient derived secondary GBM (NCH551b). Furthermore, in orthotropic mIDH1 R132H glioma in mice by NCH551b, there was significant prolongation of survival with drug gavage.

However, there is an underlying assumption that greater oncometabolite D2HG production is directly related to poorer clinical outcomes in glioma. In support of this notion, a single study by Natsumeda *et al*. [36] showed gliomas with high D2HG accumulation detected by MRI had better overall survival (OS) than gliomas with low D2HG accumulation. Additionally, Chen *et al*. [37] recently proposed through retrospective review that mIDH1 gliomas are more likely to cause seizures in patients compared with wtIDH1 due to an increase in local D2HG and dysregulated glutamate equilibrium as a result of increased glutaminolysis, which complicates clinical course.

While it appears that the D2HG level remains the forefront measure of mIDH1 severity from a biochemistry perspective, clinical evidence is required to validate this concept therapeutically. The authors note that currently, a Phase II trial examining an IDH1 inhibitor IDH305 in grade II or III glioma with any mIDH1 subtype (NCT02977689, ClinicalTrials.gov), as well as a Phase I trial examining an IDH1 peptide vaccine in grade III and IV glioma with mIDH1 R132H subtype (NCT02454634, ClinicalTrials.gov), are currently recruiting. The results of the Phase I trial studying IDH305 indicate anecdotal merit in its safety profile with glioma at the least [38]. The results of these clinical studies will be able to demonstrate more conclusively how prognostic D2HG is in glioma *via* inhibition of its production.

**Figure 2.**
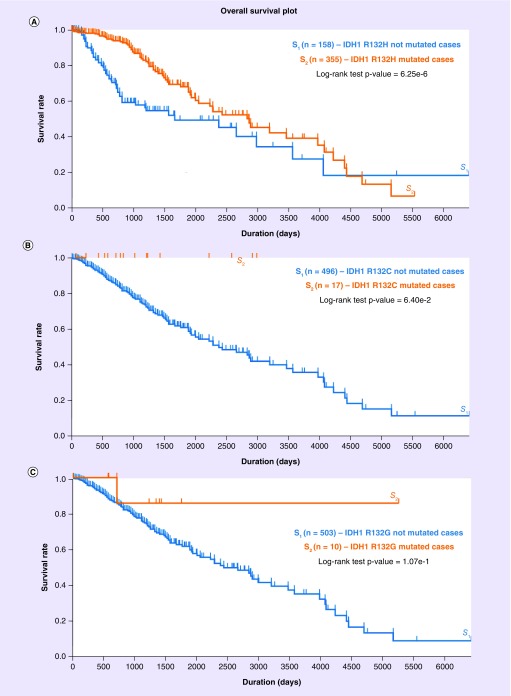
**Overall survival curves of lower-grade glioma patients with and without the mutated isocitrate dehydrogenase 1 subtype.** **(A)** R132H (log-rank test; p < 0.001), **(B)** R132C (p = 0.064) and **(C)** R132G (p = 0.107) as per the most updated TCGA. These results are derived from the publicly available TCGA-LGG database *via* the Genomic Data Commons Data Portal accessible at https://portal.gdc.cancer.gov TCGA-LGG: The Cancer Genome Atlas Lower-Grade Glioma.

Currently, there is limited survival data by mIDH1 R132 subtype, due to the novel nature of this question. Of the three most common subtypes R132H/C/G, data according to The Cancer Genome Atlas [10] indicates there to be significant difference in OS advantage between the presence and absence of R132H only in LGG, and not with R132C/G (Figures 2A–C). This implies that some mIDH1 subtypes may not be prognostically beneficial in terms of survival compared with the others, highlighting a possible difference in survival outcome based on substituting the AA within these mutations. However, all these results are currently severely limited by a number of factors which include: extremely small sample sizes, in particular for nonhistidine substituting subtypes (non-R132H); unstandardized management strategies; no control for other R132 mutations that may be present instead; and no control for other genetic mutations, such as TP53 and 1p/19q codeletion.

It is interesting to note that the rankings of arginine (wtIDH1) and histidine (R132H, the most common mIDH1 subtype) among all AAs in terms of various properties are more similar to each other when compared with the other glioma mIDH1 substituting AAs reported in the literature (Table 2). Thus, another potential classification approach to commence preliminary stratification of mIDH1 subtypes would be based on their substituting AA in relation to wtIDH1 arginine. Given, worse clinical prognosis is associated with wtIDH1 in glioma, perhaps AAs dissimilar to arginine in the mIDH1 subtype positively affect survival more than AAs similar to arginine.

**Table 2.** **Ranking of amino acids that substitute in glioma mutated IDH1 subtypes observed in the literature among all 20 standard amino acids.**

**Amino acid at site 132**	**Least hydrophobic**	**Greatest van der Waals volume**	**Highest isoelectric point**	**Greatest molecular weight**	**Greatest potential H bonds**
R, arginine	1st	2nd	1st	3rd	1st

H, histidine	7th	9th	3rd	5th	= 6th

C, cysteine	12th	17th	17th	14th	= 12th

G, glycine	13th	20th	6th	20th	= 12th

S, serine	8th	18th	13th	18th	= 6th

L, leucine	17th	7th	8th	13th	= 12th

P, proline	10th	16th	9th	16th	= 12th

V, valine	3rd	12th	4th	12th	= 12th

Arginine (R) and histidine (H) are highlighted as they represented the prototypical amino acid at IDH1 site 132 in the wtIDH1 and mIDH1 respectively.

H bond: Hydrogen bond; mIDH1: Mutated isocitrate dehydrogenase 1; R132: Arginine at site 132; wtIDH1: Wild-type isocitrate dehydrogenase 1.

Data taken from [39].

Further rigorous and prospective studies are needed to ascertain just how influential the substituting AA of different R132 mIDH1 subtypes with respect to clinical course in specific glioma. Unfortunately, this is not facile to execute. The difficulties faced include accounting for the rare nature of all non-R132H mIDH1 subtypes (<10%) and discerning prognostic change in outcomes of a disease with an already poor prognosis. A reasonable initial approach to evaluate the clinical consequences of mIDH1 by subtype in glioma would be to study outcomes of mIDH1 subtypes R132H versus non-R132H [40]. Collaboration between institutions correlating mIDH1 subtypes to clinical outcomes is highly encouraged to address the issues of cohort size and improve standardization of management.

## Conclusion

It is not inconceivable that one day the IDH1 mutation subtype of glioma may influence the stratification of current management, particularly LGG and secondary glioblastoma. Furthermore, insights into AA properties that exert influence on enzyme kinetics may provide avenue for prognostic refinement and therapeutic innovation. As we continue to develop a more comprehensive understanding of phenotypic variation between the different IDH1 mutations in glioma, this potential of subtype stratification by substituting AA will be realized.

## Future perspective

The authors speculate that in the future mIDH1 subtypes will influence glioma management, by facilitating further clinical stratification based on relative oncometabolite D2HG production. Within mIDH glioma group, those subtypes with substituting AAs that are more similar to arginine of the wtIDH1 may need to be treated by more novel, aggressive therapy, typical to that of wtIDH1 glioma and its inferior outcomes compared with mIDH1. Those subtypes with substituting AAs are more dissimilar to arginine and may represent the mIDH1 subtype that exhibits a better response to current therapy, typical of mIDH1 glioma. These conclusions rely on the assumption that D2HG is truly oncogenic, and it would be of great benefit to the field of neuro-oncology if this can be substantiated by robust cellular modelling in the future.

Furthermore, clinically what remains uncertain is, to what effect inhibiting mIDH1 to reduce D2HG production will have on improving OS outcome. As has been noted in much of the literature, the presence of mIDH1 likely confers a survival advantage compared with wtIDH1. Hence inhibiting what confers a survival advantage is counterintuitive to a certain degree and needs to be investigated. The alternative to this would be to promote the mutation, which at present, is not a consideration. Although, the authors posit that in the case of mIDH1, there is dysregulation of the NAPDH-GSH oxidation protection system in the cell which may render the cancer cells more vulnerable to conventional radiation therapy. How this aspect is used to clinically benefit the patient requires further careful evaluation. Nonetheless, the results of ongoing clinical studies targeting mIDH1 will be able to provide insight into how effective this approach truly is.

## References

[1] Buckner J, Giannini C, Eckel-Passow J (2017). Management of diffuse low-grade gliomas in adults [mdash] use of molecular diagnostics. *Nat. Rev. Neurol.*.

[2] Sanson M, Marie Y, Paris S (2009). Isocitrate dehydrogenase 1 codon 132 mutation is an important prognostic biomarker in gliomas. *J. Clin. Oncol.*.

[3] Yan H, Parsons DW, Jin G (2009). *IDH1* and *IDH2* mutations in gliomas. *N. Engl. J. Med.*.

[4] Zhu H, Zhang Y, Chen J (2017). *IDH1* R132H mutation enhances cell migration by activating AKT-mTOR signaling pathway, but sensitizes cells to 5-FU treatment as NADPH and GSH are reduced. *PLoS ONE*.

[5] Huang LE, Cohen AL, Colman H, Jensen RL, Fults DW, Couldwell WT (2017). IGFBP2 expression predicts IDH-mutant glioma patient survival. *Oncotarget*.

[6] Turcan S, Rohle D, Goenka A (2012). *IDH1* mutation is sufficient to establish the glioma hypermethylator phenotype. *Nature*.

[7] Pusch S, Schweizer L, Beck A-C (2014). D-2-Hydroxyglutarate producing neo-enzymatic activity inversely correlates with frequency of the type of isocitrate dehydrogenase 1 mutations found in glioma. *Acta Neuropathol. Commun.*.

[8] Mellai M, Piazzi A, Caldera V (2011). *IDH1* and *IDH2* mutations, immunohistochemistry and associations in a series of brain tumors. *J. Neurooncol.*.

[9] Hartmann C, Meyer J, Balss J (2009). Type and frequency of *IDH1* and *IDH2* mutations are related to astrocytic and oligodendroglial differentiation and age: a study of 1010 diffuse gliomas. *Acta Neuropathol.*.

[10] Grossman RL, Heath AP, Ferretti V (2016). Toward a shared vision for cancer genomic data. *N. Engl. J. Med.*.

[11] Balss J, Meyer J, Mueller W, Korshunov A, Hartmann C, Von Deimling A (2008). Analysis of the *IDH1* codon 132 mutation in brain tumors. *Acta Neuropathol.*.

[12] Gravendeel LAM, Kloosterhof NK, Bralten LBC (2010). Segregation of non-p.R132H mutations in *IDH1* in distinct molecular subtypes of glioma. *Hum. Mutat.*.

[13] Watanabe T, Vital A, Nobusawa S, Kleihues P, Ohgaki H (2009). Selective acquisition of *IDH1* R132C mutations in astrocytomas associated with Li-Fraumeni syndrome. *Acta Neuropathol.*.

[14] Watanabe T, Nobusawa S, Kleihues P, Ohgaki H (2009). *IDH1* mutations are early events in the development of astrocytomas and oligodendrogliomas. *Am. J. Pathol.*.

[15] Rosiak K, Smolarz M, Stec WJ (2016). IDH1R132H in neural stem cells: differentiation impaired by increased apoptosis. *PLoS ONE*.

[16] Altieri R, Zenga F, Ducati A (2017). Tumor location and patient age predict biological signatures of high-grade gliomas. *Neurosurg. Rev.*.

[17] Paldor I, Pearce FC, Drummond KJ, Kaye AH (2016). Frontal glioblastoma multiforme may be biologically distinct from non-frontal and multilobar tumors. *J. Clin. Neurosci.*.

[18] Paldor I, Drummond KJ, Kaye AH (2016). IDH1 mutation may not be prognostically favorable in glioblastoma when controlled for tumor location: A case-control study. *J. Clin. Neurosci.*.

[19] Matteo DA, Grunseth AJ, Gonzalez ER (2017). Molecular mechanisms of isocitrate dehydrogenase 1 (*IDH1*) mutations identified in tumors: the role of size and hydrophobicity at residue 132 on catalytic efficiency. *J. Biol. Chem.*.

[20] Lee SM, Koh HJ, Park DC, Song BJ, Huh TL, Park JW (2002). Cytosolic NADP(+)-dependent isocitrate dehydrogenase status modulates oxidative damage to cells. *Free Radic. Biol. Med.*.

[21] Reitman ZJ, Duncan CG, Poteet E (2014). Cancer-associated isocitrate dehydrogenase 1 (IDH1) R132H mutation and d-2-hydroxyglutarate stimulate glutamine metabolism under hypoxia. *J. Biol. Chem.*.

[22] Ohka F, Ito M, Ranjit M (2014). Quantitative metabolome analysis profiles activation of glutaminolysis in glioma with IDH1 mutation. *Tumour Biol.e*.

[23] Macdonald MJ, Brown LJ, Longacre MJ, Stoker SW, Kendrick MA, Hasan NM (2013). Knockdown of both mitochondrial isocitrate dehydrogenase enzymes in pancreatic beta cells inhibits insulin secretion. *Biochim. Biophys. Acta*.

[24] Metallo CM, Gameiro PA, Bell EL (2011). Reductive glutamine metabolism by IDH1 mediates lipogenesis under hypoxia. *Nature*.

[25] Dang L, White DW, Gross S (2009). Cancer-associated *IDH1* mutations produce 2-hydroxyglutarate. *Nature*.

[26] Dang L, Su SM (2017). Isocitrate dehydrogenase mutation and (R)-2-hydroxyglutarate: from basic discovery to therapeutics development. *Annu. Rev. Biochem.*.

[27] Dang L, Jin S, Su SM (2010). *IDH1* mutations in glioma and acute myeloid leukemia. *Trends Mol. Med.*.

[28] Zhao S, Lin Y, Xu W (2009). Glioma-derived mutations in *IDH1* dominantly inhibit IDH1 catalytic activity and induce HIF-1alpha. *Science (New York, NY)*.

[29] Jalbert LE, Elkhaled A, Phillips JJ (2017). Metabolic profiling of *IDH* mutation and malignant progression in infiltrating glioma. *Sci. Rep.*.

[30] Xu X, Zhao J, Xu Z (2004). Structures of human cytosolic NADP-dependent isocitrate dehydrogenase reveal a novel self-regulatory mechanism of activity. *J. Biol. Chem.*.

[31] Pietrak B, Zhao H, Qi H (2011). A tale of two subunits: how the neomorphic R132H *IDH1* mutation enhances production of alphaHG. *Biochemistry*.

[32] Louis D, Ohgak IH, Wiestler O, Cavenee W (2016). *World Health Organization Histological Classification of Tumours of the Central Nervous System*.

[33] Davis M, Pragani R, Popovici-Muller J (2010). ML309: a potent inhibitor of R132H mutant *IDH1* capable of reducing 2-hydroxyglutarate production in U87 MG glioblastoma cells. *Probe Reports from the NIH Molecular Libraries Program*.

[34] Rohle D, Popovici-Muller J, Palaskas N (2013). An inhibitor of mutant *IDH1* delays growth and promotes differentiation of glioma cells. *Science (New York, NY)*.

[35] Pusch S, Krausert S, Fischer V (2017). Pan-mutant *IDH1* inhibitor BAY 1436032 for effective treatment of IDH1 mutant astrocytoma *in vivo*. *Acta Neuropathol.*.

[36] Natsumeda M, Igarashi H, Nomura T (2014). Accumulation of 2-hydroxyglutarate in gliomas correlates with survival: a study by 3.0-tesla magnetic resonance spectroscopy. *Acta Neuropathol. Commun.*.

[37] Chen H, Judkins J, Thomas C (2017). Mutant *IDH1* and seizures in patients with glioma. *Neurology*.

[38] Dinardo CD, Schimmer AD, Yee KWL (2016). A Phase I study of IDH305 in patients with advanced malignancies including relapsed/refractory AML and MDS that harbor IDH1R132H mutations. *Blood*.

[39] National Center for Biotechnology Information (2016). Amino Acid Explorer. http://www.ncbi.nlm.nih.gov/Class/Structure/aa/aa_explorer.cgi.

[40] Gravendeel LA, Kloosterhof NK, Bralten LB (2010). Segregation of non-p.R132H mutations in IDH1 in distinct molecular subtypes of glioma. *Hum. Mutat.*.

